# The Role of Oxidative Stress in Cardiovascular Aging and Cardiovascular Diseases

**DOI:** 10.3390/life11010060

**Published:** 2021-01-15

**Authors:** Carmine Izzo, Paolo Vitillo, Paola Di Pietro, Valeria Visco, Andrea Strianese, Nicola Virtuoso, Michele Ciccarelli, Gennaro Galasso, Albino Carrizzo, Carmine Vecchione

**Affiliations:** 1 Department of Medicine, Surgery and Dentistry “Scuola Medica Salernitana”, University of Salerno, Baronissi, 84081 Salerno, Italy; carmine.izzo93@gmail.com (C.I.); paolovitillo@gmail.com (P.V.); pdipietro@unisa.it (P.D.P.); valeriavisco1991@libero.it (V.V.); andreastrianese@hotmail.com (A.S.); n1virtuoso@gmail.com (N.V.); mciccarelli@unisa.it (M.C.); ggalasso@unisa.it (G.G.); albino.carrizzo@gmail.com (A.C.); 2 Department of Angio-Cardio-Neurology, Vascular Physiopathology Unit, IRCCS Neuromed, 86077 Pozzilli, Isernia, Italy

**Keywords:** aging, oxidative stress, cardiovascular diseases, molecular mechanisms

## Abstract

Aging can be seen as process characterized by accumulation of oxidative stress induced damage. Oxidative stress derives from different endogenous and exogenous processes, all of which ultimately lead to progressive loss in tissue and organ structure and functions. The oxidative stress theory of aging expresses itself in age-related diseases. Aging is in fact a primary risk factor for many diseases and in particular for cardiovascular diseases and its derived morbidity and mortality. Here we highlight the role of oxidative stress in age-related cardiovascular aging and diseases. We take into consideration the molecular mechanisms, the structural and functional alterations, and the diseases accompanied to the cardiovascular aging process.

## 1. Introduction

One of the main risk factors for cardiovascular diseases (CVD) is aging [1]. CVDs and aging go hand in hand, both from a prevalence perspective and from a cause-and-effect point of view [2]. Aging is inevitable and the role of oxidative stress in this process is undeniable [3]. By 2030, one-fifth of the world population will be over 65 and CVDs rate will rise exponentially [4]. The rise of concomitant comorbidities, such as metabolic syndrome and diabetes will furthermore weight on the sustainability of health care cost and management [5]. The understanding of the mechanisms behind oxidative stress age-related CVD has led to an overturning on prevention [6]. Seeing cardiovascular aging as a modifiable risk factor opens the horizons to prevention in older patients and to a possible beneficial effect on reducing, morbidity, disability, and health care costs [7].

Many CVDs have been associated, in part or completely, with oxidative stress [8,9,10,11]. Intracellular excess production of reactive oxygen species (ROS) is mainly driven by mitochondrial metabolism and by the NOX family enzymes [12]. Although ROS are essential for various cellular functions, DNA, proteins, and lipids are affected negatively by oxidative stress giving various degrees of alterations on signaling pathways, gene expression and overall cell functioning [13,14,15]. Oxidative stress increases throughout the aging process, also interacting intensively with the longevity network and the longevity genes [16]. Overall, this is the oxidative stress theory of aging based on the hypothesis that oxidative based-damage accumulation leads to cellular senescence [17].

This review will shed some light on the role of oxidative stress age-related CVD, first from a molecular and genetic standpoint and then from a pathophysiology and clinical standpoint.

## 2. Behind the Scenes of Cardiovascular Aging

### 2.1. Sources of Oxidative Stress and Oxidative Damage

The oxidative stress theory of aging is based on the hypothesis that age-related loss of functions of tissues is due to the accumulation of oxidative damage to macromolecules (DNA, proteins, and lipids) [17]. These damages are mediated by free radicals formed in redox reactions where a reductant product is oxidized donating an electron to an oxidant. Free radicals, like reactive species of oxygen (ROS) and nitrogen (RNS), are generated as products of redox reaction with one or more unpaired electrons in external shell [18]. Our biologic systems have mechanisms to neutralize oxidative stress, but these decline with aging process and this contributing to imbalance between generation and clearance of ROS, increasing ROS-mediated damage [19]. The main endogenous process who generates ROS is oxidative phosphorylation [18]. This is a compound of mitochondrial reactions during electrons from nicotinamide adenine nucleotide (NADH) and flavin adenine dinucleotide (FADH) are processed via four mitochondrial enzymes with formation of adenosine triphosphate (ATP) from adenosine diphosphate (ADP). Electrons lost during the process form superoxide radical that lead functional impairment of tissues damaging macromolecules [20]. Furthermore, superoxide can react with nitric oxide with production of peroxynitrite, a powerful membrane permeable oxidant that inactivate several enzymes though nitration. Superoxide anion can also convert in hydrogen peroxide trough superoxide dismutase. The hydrogen peroxide through Fenton reaction produce highly reactive radical named hydroxyl radical [21]. These ROS produced via mitochondria are the most important for cardiac tissue in consideration of 45% of hearth’s cell volume is formed by mitochondria [22]. These mechanisms are also implicated in age related damage to vasculature [23]. Mitochondrial ROS can activate some transcription factors like NF-kb and AP-1 promoting an inflammatory pathway on tissues [24]. Mitochondria derived ROS produced severe vascular disfunction mediated by the downregulation of Nrf2 (an antioxidant pathway against oxidative stress), the reduction of NO bioavailability, and the increasing production of vasoconstrictor molecules [25]. The oxidation process cause rupture of mitochondrial membranes with the release of pro apoptotic molecules to the cytosol increasing plaque cell apoptosis and plaque fragility, stimulating smooth muscle cell proliferation, and increasing leukocyte adhesion and inflammatory response [16]. ROS generated from NADPH oxidases during inflammatory responses plays an important role in free radical endogenous production [26]. These transmembrane enzymes oxidize NADPH producing NADP and H_2_O_2_ whose can diffuse through membranes [27]. The importance of ROS in the host immune response is demonstrated by the fact that people with an inherited deficiency in NOX2 develop chronic granulomatous disease and are enabled to respond in front of common infections. A basal concentration of ROS is indispensable for cellular homeostasis, on the other hand excessive levels of ROS cause damage to cellular macromolecules and it is one of the main mechanisms implicated in aging and CVDs. Endothelial disfunction mediated by NADPH oxidation is suggest by the findings of an improved endothelial vasodilation after NADPH oxidase inhibition with apocynin [28].

Another mechanism of ROS production is NOS uncoupling, caused by BH4 and L-arginine deficiency [29], with consequent increasing production of superoxide anion [30]. Both eNOS and iNOS are subject of uncoupling in absence of the same substrates [31]. Mitochondrial ROS, activation of NADPH oxidases and uncoupling of NOS establish a positive feedback each other causing an increment of ROS concentration [32]. Other sources of exogenous ROS and RNS are air, water, tobacco, alcohol, heavy or transition metals, drugs (e.g., cyclosporine, gentamycin, and others), industrial solvents, cooking (e.g., smoked meat, waste oil, and fat), and radiation, which inside the body are metabolized in free radicals [33].

ROS and RNS, whether they are endogenous or exogenous, cause oxidative modification of each of the major cellular macromolecules (carbohydrates, lipids, proteins, and DNA). Protein carbonyl is formed by Fenton reaction of oxidants with lysine, arginine, proline, and threonine residues of the protein side chains [34]. Carbonyl groups may also derive from the binding of aldehydic lipid oxidation products to lysine, cysteine, or histidine residues called Michael-addition reactions [35]. Reactions between RNS and tyrosine residues free or within polypeptide sequences induce the formation of nitrotyrosine [35]. Low-density lipoproteins (LDL) are the major carriers of cholesterol to body tissues. RNS and ROS have a central role in atherosclerosis development oxidizing low density lipoproteins. In fact, ox-LDL enter the subendothelial spaces and lead the atherosclerosis process forming atheroma, as explained previously. Important targets of lipid peroxidation are poly-unsaturated fatty acids (PUFAs), in particular linoleic and arachidonic acids, trough hydroxyl, and peroxyl radicals. Depending on the type of PUFAs undergoing lipid oxidation, some different reactive aldehydes are produced, such as trans-4-hydroxy-2-nonenal, malondialdehyde, and isoprostanes [36]. The amino groups of lysine and arginine react with the carbonyl groups of carbohydrates in glycoxidation process, results in advanced glycation end products (AGE). Major AGEs are hydroimidazolone, pentosidine, and glucosepane. Oxidative stress is also well known to be implicated in negatively effects on telomeres loss. Telomeres are structures of repeating nucleotides at the end of eukaryotic chromatids that shorten replication after replication. This mechanism protects important genetic data lost by incomplete replication. Telomeres length is determined from unknown genetic factors, but a lot of conditions are associated with their shortening like insulin resistance, hypertension, and oxidative stress [37]. When shortened to critical length telomeres lead cells towards a senescence state. The telomeres deterioration is associated with cardiovascular decline, congestive heart failure and peripheral vascular disease [38]. Advanced biological age is characterized by the accumulation of epigenetic changes that can be correlated with CVDs [39].

The precise mechanism of oxidative stress-induced aging is still not clear. It is well established that elevated ROS levels lead to cellular senescence. This mechanism stops cellular proliferation in response to damages occurring during replication. Senescent cells acquire an irreversible senescence-associated secretory phenotype involving secretion of soluble factors (interleukins, chemokines, and growth factors), degradative enzymes like matrix metalloproteases (MMP), and insoluble proteins/extracellular matrix (ECM) components [40,41]. ROS induces cellular senescence activating many mechanisms, the main are:

- production of IL-1α leading to a proinflammatory state, which increases nuclear factor kappa-β (NFκβ) activity [40];
- induction of MMPs expression, which is associated with age-related and chronic diseases such as cancer, Alzheimer’s, atherosclerosis, osteoarthritis, and lung emphysema [40];
- inhibition of FOXO (Forkhead box) proteins activity, which is involved in insulin/insulin-like growth factor-1-mediated protection from oxidative stress [40];
- reduction of sarco/endoplasmic reticulum Ca^2+^-ATPase activity leading to cardiac senescence;
- inhibition of sirtuins activity leading to an increased production of ROS and RNS by SOD inhibition, a proinflammatory state by preventing their inhibition of tumor necrosis factor alpha (TNFα) and NFκB, and tumorigenic effect by preventing their inhibitory effect on c-Jun and c-Myc [42];
- regulation of p16INK4a/pRB and p53/p21 pathways leading to senescence [40].

### 2.2. Aging, Oxidative Stress and Cellular Dysfunction

Age-dependent oxidative stress cellular dysfunction is a condition determined by various intrinsic factors. The heart cell, the cardiomyocytes, exert a high aerobic metabolism in order to guarantee their functions. In this scenario cellular metabolism is guaranteed by mitochondrial function [43]. Mitochondria occupy 30% of total cardiomyocyte volume, this guarantees the high energy demands of the heart. Mitochondrial oxidative phosphorylation fueled by catabolism of lipids and carbohydrates is the main source of ATP for heart function. The Krebs cycle is important for ATP generation but its balance and regulation are fundamental for oxidative stress management [44]. Cardiomyocytes aging is one of the main factors leading to cardiovascular diseases. Understanding the processes behind cellular dysfunction in the aging process has led to many findings in relation to mitochondrial dysfunction and its genomic instability and epigenetic regulation [45]. In fact, studies on longevity have highlighted the importance of mitochondria and oxidative stress in the senescence process [46]. As described before, free radicals are generated in different compartments of the cell. However, the main source of free radicals is mitochondria and in particular the electron transport chain located in the inner mitochondrial membrane. The continuous production of free radicals in the mitochondria leads to oxidation of macromolecules and DNA [47]. In particular, during aging nuclear and mitochondrial DNA stability is compromised [48]. Mitochondrial DNA lesions have been shown to carry mutagenic and cytotoxic affects ultimately leading to DNA replication alterations [49,50]. The genomic instability is partially prevented by DNA repair pathways in both mitochondria and nucleus [51]. Despite this DNA damage increases and accumulates during the aging process. Aged mice heart mitochondria have a threefold higher frequency of mitochondrial DNA mutation and deletions [52]. Accumulation of DNA alterations is a driving factor in aging leading to cellular loss of function. Animal models with increased mitochondrial DNA instability mediated by disruption of mitochondrial transcription factor A, showed alteration of mitochondrial morphology leading to higher rates of apoptosis and ultimately to dilated cardiomyopathy [53,54]. An early aging phenotype is characterized by high mitochondrial DNA point mutation as highlighted in mice [55,56]. New animal models have been developed to study the effect of increased mitochondrial DNA deletions in the myocardium. These models have highlighted that mitochondrial DNA dysfunction is not only associated with tissue dysfunction but also with development of premature arrhythmias [57]. Mitochondrial dysfunction and instability contribute to the aging process in other ways as is the case with calcium homeostasis and redox signaling [58,59]. Overall, the aging process and the mitochondrial alterations lead to metabolic changes. Normally up to 90% of cardiac ATP is generated by the use of long-chain fatty acid, only 10% from glucose, lactate, ketone bodies, and amino acids [60,61]. The Randle cycle highlights the substrate selection and interaction. Development of heart disease and aging has been associated with the inversion of metabolic substrate selection [61]. With aging increased glucose metabolism expression, increased glycolytic proteins and the concomitant decline in fatty acid oxidation has shown to be a premature sign of heart failure in younger individuals [62]. As we will mention afterwards, metabolic inversion and heart failure are associated with a reactive hyper-adrenergic state closely linked to impaired glucose metabolism and inevitably to insulin resistance [61].

Another important cellular dysfunction point to address is increased apoptosis during aging [63]. Accumulation of lipofuscin on lysosomes, loss of autophagic management, accumulation of non-functional damaged cell components, in particular mitochondria, leads to activation of the apoptotic pathway [64]. These changes in cardiomyocytes also increase alteration in calcium cellular handling. Calcium plays a crucial role in modulating cardiac function by modulation of myocardial contractility through a complex system of ion channels, ryanodine receptors, and sodium calcium exchangers. On the other hand, myocardial relaxation is mediated by sarcoplasmatic/endoplasmatic reticulum calcium-ATPase (SERCA2a) which seizes calcium to the sarcoplasmic reticulum [65]. The importance of cellular SERCA2a function has been highlighted in oxidative stress and aging. In particular, SERCA2a impairment and consequent increase in calcium stagnation in cytoplasm causes diastolic relaxation impairment [3]. Moreover, animal models by the same mechanism of prolonged cellular contraction showed alterations in myosin heavy chain expression [66]. These changes influence negatively cardiomyocytes contractile and electrical efficiency [66]. Alteration of the contraction and relaxation coupling of cardiomyocytes is at the base of the compensation mechanism that we will describe in the next chapters [67]. In fact, we will see how depletion of cardiomyocytes and inadequate contraction efficiency leads to cardiac hypertrophic remodeling and not only [68].

### 2.3. Aging in Cardiac Structural and Patho-Physiological Changes

Cardiac structural modifications due to aging bring about various functional and adaptive consequences in heart capacity [69]. Cardiac aging causes fibrosis, left ventricle hypertrophy, diastolic dysfunction, and filling ultimately leading to reduction in cardiac overall compliance and ejection fraction output [70,71]. Gene expression in aging human hearts highlights a shift in cellular capacity. Upregulation of genes for sarcomere and cytoskeletal proteins and downregulation of proteasome components expression reflects the process of myocyte hypertrophy and decreased in protein turnover [72]. This is accompanied by downregulation in Troponin T, SERCA2, and alpha-MHC with a switch towards beta-MHC, with a decrement in contractile efficiency [73]. Mitochondrial disfunction in mice aging cardiomyocytes is an important source of oxidative stress [74]. The inefficiency of aging rat mitochondria is characterized by increase in size, reduction in number of cristae with an overall reduction in ATP production per cell [75]. The high-rate production of reactive oxygen species leads to a vicious cycle [76,77]. In fact, in order to maintain functional demands the aging heart undergoes a process of hypertrophy [78]. This adaptation allows the heart to respond to increase in pressure demand in consequence to vascular stiffness [79]. However, this mechanism fails when heart demands exceed bringing about heart failure [78]. Cardiac hypertrophy in human aging-heart is characterized by increase in cardiomyocyte size with asymmetric left ventricle ventricular wall thickening mainly of the intraventricular septum [80]. These alterations cause alterations in human heart geometry and shape initially granting increase in contractility but on the long run causing oxygen impairment and reduction in contractility [81]. Aging in both mice models and humans is associated with apoptosis and loss of myocytes with a rate of about 45 million per year compensated by increase in single myocyte median volume [82,83,84,85]. The overload of the remaining myocytes is an additional reason for compensatory hypertrophy [86]. In addition, the loss of vascular elasticity and the increase in vascular stiffness increases the mechanical load of the aging heart accelerating the way to heart failure [87]. Age-related left ventricle hypertrophy in humans occurs independently of underlining story of hypertension or other causes [88]. Overall, a healthy human aging heart with preserved ejection fraction (EF) shows no change in stroke volume, heart rate, and cardiac output [81]. The main difference is highlight in diastolic function, in fact, the capacity of the ventricle to receive blood results altered by the reduction in ventricle wall elastic compliance [89]. Hemodynamically this can be seen in a reduction in early diastolic filling with an increase in tele systolic filling with an inversion of the E/A parameter and an increase in E/e’ better seen in echocardiographic studies [90,91,92]. This compensatory system present at rest however is not efficacious in case of cardiac demands. The aging-heart in fact compensates the contractile and diastolic function reduction with increase in peak heart rate [93]. Another important age-related alteration in animal model hearts is the calcification of the valves associated with proliferation of fibroblasts and deposition of collagen [94,95,96]. The cardiac extracellular matrix in the aging heart fills with glycoproteins, proteoglycans, glycosaminoglycans, integrins, and collagen [97]. Interesting is the switch from collagen type III to type I, seen in children, young adults, and elderly, which is less distensible and stiffer [98]. This also effects negatively the propagation of the electrical signals of the myocytes and of the myofibrillar bundles giving arrhythmias [99,100].

### 2.4. Endothelial Dysfunction

Age-dependent endothelial dysfunction is a multifactorial disease characterized by vascular endothelial cell structural and functional deterioration [28]. The reducing property of vasodilatation of the human aging endothelium is due to the loss of the homeostasis between vasoconstrictor and vasodilator molecules, and is associated with an increased mobility, and mortality [101,102,103]. In fact, the enzyme cyclooxygenases (COXs) are able to produce both contractile and vasodilators factors like TXA2, prostacyclin, and prostaglandin I2 (PGI2), respectively [104]. In the elderly, the loss of prostacyclin endothelium-dependent vasodilatation [105,106] and an increasing production of TXA2 by COX, ultimately causes an increasing endothelium-dependent contraction. As seen in eNOS knockout mice, the COX pathway is heavily influenced by the interaction with NO pathway, this is one of the main factors associated with cardiovascular health and longevity [107,108]. NO is a vasodilator, produced by NO synthase, an enzyme with three isoforms: neuronal NOS (nNOS), inducible NOS (iNOS), and endothelial NOS (eNOS). eNOS is the responsible of the vasodilatation in response to artery sheer-stress derived. In aging endothelium the eNOS activity reduction [109] reduces NO bioavailability [110]. Furthermore, in an aging endothelium high reaction rate between NO and superoxide anion results in NO activity reduction and generate peroxynitrite. This RNS can penetrate the cell membrane and react with macromolecules such as lipids and DNA with consequent premature aging [111]. Furthermore, in diabetic humans peroxynitrite seems to be a ROS production promoter factor establishing a vicious cycle [112]. This inflammatory pathway self-induced by the endothelium seems have a link with the vascular smooth cell expression of MMP-2 and MMP-9 with a devasting effect on vessels remodeling. In fact, in diabetic patients the impaired vasodilatation mechanism is followed by arterial stiffness thus indicating a link by endothelial disfunction and morphologic arterial changes [113]. Macroscopically aging vasculature is characterized by large elongated and tortuous arteries with a thickened wall and an enlarged lumen [114]. In the elderly, the reduction of endothelial cells turnover contributes greatly to endothelial dysfunction. This can be demonstrated by the decreased number and malfunction of the endothelial progenitor cells in aged humans [115]. The turnover of the endothelial cell is essential to preserve integrity, maintenance, and regeneration of the endothelial layer. This lack reduce vasodilatation/contraction endothelium mediated and reduced capacity in repair of injured arteries [116,117]. Moreover, in aging vasculature is evident the reduction of elastin and an increase of collagen fibers with the consequent reduction of the skill to transfer the blood flow pulse wave caused by the loss of the distensibility [118]. To guarantee a structural integrity collagen and elastin are kept together by an enzymatic cross-linking but in elderly vessels this link is mediated by a non-enzymatic glycation which lead an inflammatory response and a low resistance to the MMP activity, increasing fibrosis of tissues [68,119]. The mechanisms described earlier cause a negative hemodynamic cycle characterized for an increase of the peripheral resistance and pulse pressure with cardiovascular and systemic devasting consequences.

### 2.5. Aging and Autonomic Nervous System Adaptations

With aging an increasing plasma catecholamine concentrations and sympathetic tone of autonomic nervous system (ANS) [120,121] could impair adaptivity of elderly individuals to the environment [122]. Aging has been implicated in the impairment of alfa and beta-adrenergic receptor sensitivity and vascular responsiveness [102,123] with a predominance of the alfa tone in muscle vasculature. In fact, in older women we can observe a higher decrease of arterial blood pressure in front of younger ones after an autonomic blockade [103]. Age-related alterations in ANS influence blood pressure, cerebral blood flow, bladder function, and heart rate variability (HRV) [79,124,125]. HRV is an indicator of arrhythmic complications and a strong predictor of mortality and sudden death [126]. Nocturnal reduction of parasympathetic activity in elderly individuals is the result of a low vagal hearth control due to the prevalence of the adrenergic tone of SNA. The increase in heart rate combined with the HRV reduction is due to the degeneration of cardiac autonomic function during aging [127] and increases the incidence of cardiovascular events [128]. Active lifestyle is important for the health of the elderly. Intensive physical exercise, increasing muscle mass and fatigue resistance [129], could minimize autonomic dysfunction in aging with training [130]. A physical training regime could improve adaptations of the autonomic function [131]. Although intensive exercise seems to have benefit for older individuals, there is a need for further studies to realize a regimen of exercise programs. The ANS abnormalities were thought to be a common underlying pathophysiology of CVDs such as hypertension and heart failure [132]. Another issue due to the impairment between the two ANS deregulation is atrial fibrillation, the most common arrhythmia in older people [133,134]. Denervation of ANS has been shown to be efficient against AF [135]. Patients with AF had reduction in cardiac performance due to the loss of the atrial contraction in ventricular filling.

### 2.6. Genetic and Epigenetic Regulation on Cardiovascular Aging and Oxidative Stress

In addition to being a common denominator to many chronic disease manifestations, oxidative stress-induced endothelial dysfunction is the key mechanism linking aging to increased risk of clinical cardiovascular disease and death [31]. As we age, the continuous imbalance between the generation of ROS—which increases progressively—and the ability of endogenous anti-oxidant defense mechanisms—which becomes increasingly reduced—results in reduced NO bioavailability and impaired vasodilatory function [136,137]. Longitudinal studies conducted in long-lived and very long-lived cohorts, including “centenarians” have demonstrated the undeniable role of genetic variations in the regulation of the oxidative stress response in aging [138,139,140], referred to as the capacity of triggering various regulatory processes in response to oxidative stress. Among the antioxidant enzymes, Soerensen and colleagues [140] showed that a major role in longevity seems to be attributed to manganese superoxide dismutase (MnSOD) and glutathione peroxidase 1 (GPX1), indicating that variation in these genes may affect human life span.

In age-related cardiovascular diseases, a prominent role of sirtuins has also been thoroughly explored. Sirtuins (Sirt) belong to class III histone deacetylases that have been associated with aging for their nicotinamide adenine dinucleotide (NAD+)-dependent enzymatic activity. Seven sirtuin family members have been identified so far, denoted as Sirt1-Sirt7, each of which displays differential subcellular localizations: Sirt1, 6, and 7 are localized in the nucleus, Sirt2 is cytosolic, Sirt3 to Sirt5 are found in the mitochondrial compartment, the major source of intracellular ROS [141]. Many existing studies have demonstrated a protective role of the sirtuins against both accelerated vascular aging and atherosclerosis [142,143,144,145,146,147]. For instance, Sirt1 deficiency increases oxidative stress, inflammation, and foam cell formation and induced the senescence of endothelial as well as vascular smooth muscle cells. In support of this notion, using human vascular smooth muscle cells (VSMC) isolated from donors (from 12 to 88-year-old subjects), Thompson and colleagues [148] demonstrated an inverse correlation between the endogenous Sirt1 protein expression and the donor age. In an independent study conducted in 3763 subjects, the authors showed that homozygous minor allele genotypes within rs2841505 (Sirt5) and rs107251 (Sirt6) negatively influenced survival, whereas homozygous minor allele genotypes within rs511744 (Sirt3) were associated with an increased lifespan [149]. Furthermore, while partial Sirt1 deletion in atherosclerotic mice enhanced atherogenesis [150], Sirt1 overexpression reduced atherosclerotic plaque formation in *ApoE^−/−^* mice following 10 weeks of high-fat diet feeding [151]. In an attempt to investigate the molecular mechanisms behind the role of Sirt1 in inducing lifespan expansion, deletion of Beclin 1/Autophagy protein (Atg) 6 was found to abrogate longevity in C. Elegans [152]. In fact, besides being implicated in a variety of cellular processes, including energy metabolism cell stress response and cell/tissue survival, recent studies have also identified a role for sirtuins in the regulation of autophagy, a cellular housekeeping process, which is critical for the maintenance of tissue homeostasis during aging, particularly cardiac and vascular health [153]. For example, in response to cellular stress, including caloric restriction, Sirt1 upregulated the expression and directly deacetylated several Atg proteins, including Atg7 and Atg8, thereby inducing autophagy [154]. Likewise, Sirt1-null mice have increased basal acetylation of autophagic proteins and a reduced autophagy function [154]. In addition, knockdown of Sirt1 prevented the induction of autophagy by its indirect activator resveratrol and by nutrient deprivation in human cells [152]. Acting in a distinct manner from that of Sirt1, Sirt3 also regulated autophagy both positively and negatively. In particular, it has been reported that Sirt3 overexpression activated the mammalian target of rapamycin complex 1 (mTORC1), a consistent inhibitor of autophagy. In addition, Sirt3 gene silencing promoted autophagy and protected human hepatocytes from lipotoxicity [155]. In contrast, by inducing autophagy, Sirt3 prevented ischemia-induced neuronal apoptosis in cortical neurons cells [156]. Recent findings have also reported the beneficial role for Sirt6-mediated autophagy against cardiovascular diseases [157]. Indeed, using an in vitro model, Sirt6 reduced the formation of foam cells through an autophagy-dependent pathway, thus suggesting the protective role of Sirt6 in preventing atherosclerosis [157].

The mitochondrial adaptor protein p66^Shc^ is another key determinant of aging since its genetic deletion induces stress resistance and prolongs lifespan by 30% [158]. The mammalian SHC locus encodes for three different isoforms carrying a Src-homology 2 domain. Owing to its unique N-terminal region, p66^Shc^ is the only protein playing a critical role in redox metabolism [158]. Genetic deletion of p66^Shc^ has been shown to reduce the production of free radicals in the brain, improve stroke outcome, and preserve neurological function in an ischemia-reperfusion injury model [159]. In patients with acute ischemic stroke, p66^Shc^ expression was transiently increased and correlated with short-term neurological outcome [160]. Furthermore, p66^Shc^ has also been involved in hyperglycemia-associated changes in endothelial function. In a mouse model of streptozotocin-induced diabetes, deletion of p66^Shc^ protein protected mice against oxidative stress-induced endothelial dysfunction as well as lipid peroxidation in aortic tissue [161].

By modulating the expression of several antioxidant enzymes, the proto-oncogene JunD has been suggested as a potential target to prevent ROS-driven age-related cardiovascular diseases; indeed, JunD knockout mice exhibited impaired endothelium-dependent relaxation and reduced life span [162,163]. Conversely, in vivo JunD overexpression rescued age-induced endothelial dysfunction [162]. Moreover, low levels of JunD have been reported in monocytes of old healthy subjects when compared with young individuals, and the decrease was correlated with the expression of scavenging and oxidative enzymes [162].

Bactericidal/permeability-increasing fold-containing-family-B-member-4 (BPIFB4) has recently emerged as another molecule with important role in regulating aging and longevity. Recent studies have shown the role of a polymorphic variant in the gene encoding BPIFB4 in determining exceptional longevity in 3 independent populations [164]. In addition, homozygous carriers of the so-called *longevity-associated variant (LAV)-BPIFB4* exhibited higher circulating BPIFB4 levels and increased eNOS activity in circulating mononuclear cells [165]. In line with these findings, circulating BPIFB4 levels were closely linked with the health status of long-living individuals [165]. Interestingly, *LAV-BPIFB4* gene transfer decelerated frailty progression in aged mice and homozygous *LAV-BPIFB4* haplotype inversely correlated with frailty in elderly subjects [166].

Growing evidence suggests microRNAs (miRNA) as critical regulators of aging and cardiovascular disease [167,168,169]. Acting at the post-transcriptional levels, these small non-coding RNA molecules are able to negatively regulate gene expression by inducing mRNA degradation or translational repression of their targets [170].

First discovered in *Caenorhabditis elegans*, several miRNAs have been found to both positive and negatively regulate longevity through canonical pathways during aging, including insulin signaling, heat-shock factors (HSFs), AMP-activated protein kinases (AMPKs), mitogen-activated protein kinases (MAPKs), sirtuins, target of rapamycin (TOR), and mitochondria [169,171]. Because miRNAs and aging signaling pathways are conserved across species, from nematodes to humans, studies on *C. Elegans* have provided clues to understanding mechanisms of age-related processes. For example, it has been shown that adult-specific loss of argonaute-like gene-1 activity resulted in a shorter lifespan when compared with that of wild-type [172]. Furthermore, a loss-of-function mutation in *lin-4* and gain-of-function mutants of its target *lin-14* displayed a reduced lifespan, which was rescued to a significant extent by knockdown of *lin-14* only during adulthood [173]. Van Almen et al. found decreased miR-18 and miR-19 levels in aged heart failure-prone mice when compared to age-matched controls [174]. Importantly, these findings were also confirmed in cardiac biopsies of idiopathic cardiomyopathy patients at old age, where decreased miR-18a, miR-19a, and miR-19b expression was associated with severe heart failure [174]. Together with increased ROS production, miR-21 was found to be upregulated in heart, liver, kidney, and aorta of spontaneous hypertensive rats (SHR). Interestingly, exposure to exogenous miR-21 was able to lower blood pressure levels in the SHR rats, indicating miRNAs as novel potential therapeutic target in hypertension [175]. Among the most studied miRNAs, circulating levels of miR-126, miR-130a, miR-142, miR-21, and miR-93 have been found to be associated with human aging [176]. For example, miR-21 level is increased with aging process and aging associated-diseases, but its expression level decreased in individuals aged over 80 and in centenarians, suggesting the beneficial effects of low levels of miR-21 for longevity [176]. Menghini et al. found that miR-217 is widely expressed in aged but not young endothelial cells, and that its overexpression can induce endothelial cell senescence formation, thus suggesting the involvement of miR-217 in the pathogenesis of cardiovascular diseases [177]. Finally, using PCR arrays, Smith-Vikos et al. [167] identified multiple differentially expressed microRNA in serum samples from individuals who had documented lifespans from 58 to 92. Interestingly, six of these circulating miRNAs significantly correlated with subsequent longevity, suggesting that these miRNAs may serve as useful biomarkers of human aging [167].

Along with findings from several clinical trials that have failed to show long-term improvement with antioxidants [178,179], the above studies suggest the importance of directly acting on upstream signaling rather than adopting strategies to scavenge formed ROS to delay age-related disease onset.

## 3. Aging, Oxidative Stress, and Cardiovascular Diseases

**Figure 1:**
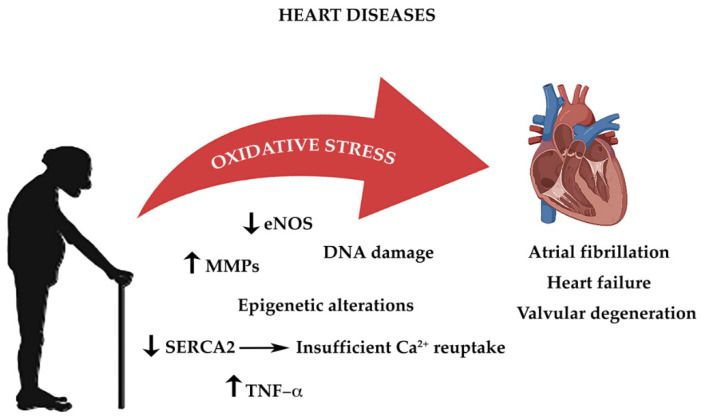
The impact of oxidative stress on age-associated heart diseases.

As highlighted above, all cells constantly undergo reactions which require transfer of electrons, in order to acquire energy [41]. These complex mechanisms, which occur principally in the mitochondria, require a constant turnover in the oxidative state [180]. The redox reactions and the reactive oxygen species (ROS) generated are highly reactive and are the main source of oxidative stress [18]. The heart’s cellular volume is made up for 45% by mitochondria [181]. Oxidative phosphorylation generates radical species, when electrons are lost from mitochondrial complexes I and II, in order to produce adenosine triphosphate (ATP) [18]. The mitochondrial pathway also characterized by formation of NADH and FADH which react with other redox compounds [182]. As cardiomyocytes and endothelial cells age, they produce more ROS. Increased production of ROS leads to functional impairment due to reduction in biological activity of nitric oxide and formation of peroxynitrite, which deactivates several free radical scavengers [23]. Another important origin of oxidative stress is aberrant Ca^2+^ reuptake, due to SERCA2 downregulation [183]. Constant high concentration of Ca^2+^ levels in the cardiomyocyte’s cytosol increases ROS production [184,185]. As highlighted before the RAAS system also plays an important role in age-related cardiac oxidative stress with direct and indirect involvement of NADPH oxidase complex and of ROS production [186,187]. Thus age-dependent increase in oxidative damage is the one of the leading causes of CVD (Figure 1) [188].

### 3.1. Atrial Fibrillation

Atrial fibrillation is the most common chronic cardiac rhythm disturbance, it affects 1–2% of the population and the chances of developing this condition increases with age, particularly after age 65 [189]. It consists in a rapid heartbeat (tachyarrhythmia) that originates in the upper chambers of the heart, called the atria, preventing them from functioning properly [190]. In such circumstances, the atria are no longer able to expel all the blood, which will remain partially inside the atria with the risk of clot formation [191]. Clot formation due to AF is one the leading causes of cerebrovascular thrombosis [192,193]. In aging the main cause of AF can be found in the hearts structural and functional alteration, as described earlier. Aging itself is the single most important risk factor for AF [194]. Among all the previously described alteration, the electrical conduction disturbances, ectopic activity leading to atrial arrhythmogenesis is atrial fibrosis and so dilation [195]. Atrial fibrosis in the human aging heart is associated with excessive accumulation of collagen fibers, in particular type I fibers, and the increase of cross-linking between fibers [196]. Oxidative stress plays an important role in extracellular matrix (ECM) turnover and metabolism. The increase of matrix metalloproteinase (MMP) in human aging hearts with atrial fibrillation has been widely highlighted [197,198]. Age-related alteration are a summation of structural and electrophysiological remodeling leading to alteration of the mechano-electrical feedback [199]. Overall, this means that the atria are no longer capable of modulating the electric functions of the heart with the induction of mechanical load on the cardiomyocytes [200]. In conclusion, aging is associated with an increase in acute-phase inflammatory cytokines an important stimulus for AF, as it directly increases arrhythmogenicity and calcium homeostasis dysregulation [201,202]. Moreover, as seen in human atrial tissues, ROS and mitochondrial disfunction influence AF due to accumulation of age-related mitochondrial DNA deletion and mutation [203,204]. Oxidative stress and inflammation in aging influence greatly both functional and structural modifications causing electrophysiological remodeling and correlated diseases [205].

### 3.2. Heart Failure

Heart failure (HF) is a complex clinical syndrome defined by the inability of the heart to supply blood in adequate quantities for the body’s actual demand or the inability to meet this demand only at ventricular filling pressures above the norm [206]. According to statistics, as the population ages and the number of patients who survive a myocardial infarction increases, the incidence of heart failure continues to rise [207]. If we refer to hemodynamics, heart failure is characterized by reduced contractility of the myocardium measured as ejection fraction (EF), this universally used parameter can actually be not very specific in identifying the cause of cardiac dysfunction. In fact, this condition can be caused by both organic and functional problems. Among the most common causes are myocardial infarction, myocardial ischemia, hypertension, valvulopathies, cardiomyopathies, metabolic diseases, and autoimmune diseases [44]. HF is the most important complication of any heart disease. In the United States of America, it is estimated that in 2006 there were more than 600,000 new cases [208]. In Italy about 5% of the general population (3,000,000 individuals) is affected by overt or asymptomatic HF [209]. Age is a very important risk factor, the incidence remains low in people between 40 and 50 years, while it rises up to 10% in people over 75 years of age [210]. The incidence of this pathology is in rapid increase due to the overall increased survival rate following acute myocardial infarction and to longer life expectancy [211]. Aging (with consequent organ degeneration) causes cardiac efficiency to decrease, amplifying the effect of any pathologies. HF is generally thought to be involved in all those deaths from aging without apparent symptoms. In fact, when cardiac contractility is progressively reduced, whatever the cause, the final effect is hypo-perfusion of the organs vital and in particular of the brain, kidneys, and liver with the progressive functional deterioration [212].

Systolic HF is characterized by the reduced performance of the left ventricle, easily identifiable in patients with a universally adopted echocardiographic parameter such as the ejection fraction (FE), which, however, can vary considerably with the different biomedical imaging methods [213]. The force of contraction of the heart is directly proportional to the conditions of the myocyte, the cell of which the heart muscle is composed: any insult that affects the myocytes is reflected on the compliance of the left ventricle and therefore on the force of contraction (the FE is generally lower than 45%) [214]. Ventricular hypertrophy is one of the adaptation mechanisms of the heart subjected to increased stress that persists for long periods of time; this attempt at correction may be a factor involved in the progression of heart failure [215]. It is certainly not the mechanism that leads to myocardial hypertrophy, it is certain that an increase in systolic wall tension in association with an increase in afterload would cause concentric hypertrophy; on the contrary, an increase in diastolic wall tension in association with an increase in preload would lead to eccentric hypertrophy [216]. In both situations the synthesis of the unit of the myocyte known as sarcomere would be stimulated: in the first case the production of sarcomeres would be stimulated in parallel and in the second in series [217]. Myocytes and their components can be damaged by inflammatory diseases (myocarditis) and by infiltrates (amyloidosis), by toxins or by drugs. The most common mechanism is certainly myocardial ischemia: with the death of myocytes, the myocardium is replaced with fibrous or connective tissue, which have no contractile properties and are similar to scars. These scars can initiate the heart remodeling process, which in turn can lead to heart failure [218]. Heart failure caused by diastolic dysfunction, like systolic dysfunction, may be symptom-free in a compensated patient [219]. What characterizes this alteration is the inability of the left ventricle to adequately relax and this is secondary to the increased stiffness of the ventricular chamber. This attitude of the heart muscle leads to a reduced ventricular filling in diastole, which translates into a reduction in output. The inability to obtain optimal relaxation leads to an increase in end-diastolic pressures, which affect the atria and pulmonary veins [220]. Diastolic dysfunction and systolic dysfunction have many causes in common, most notably older age, high blood pressure, diabetes mellitus, and left ventricular hypertrophy. We can consider separately female sex, diseases of the pericardium and hypertrophic, accumulation and infiltrative cardiomyopathies [221]. Restrictive cardiomyopathy is one of the diseases that most affect diastolic dysfunction. Restrictive cardiomyopathies are characterized by a restrictive filling and a reduced diastolic volume; they are classified into primary and secondary [217].

In the failing aging heart, oxidative stress plays an important role. Both normal aging and pathological aging human hearts show increased levels of ROS [222]. Aging and oxidative stress can be accompanied by other pathological conditions such as diabetes, endothelial dysfunction, atherosclerosis, hypertension, and degenerative diseases increasing the imbalance between ROS production and antioxidant systems [187,223]. With aging, the compensatory mechanisms do not effectively manage ROS accumulation in mice models. This generates increased oxidation of proteins, lipids, and mitochondrial DNA damage. Electron leak in mitochondria is considered one of the main sources of ROS and adenine nucleotide translocase (ANT) seems to be why [224,225,226,227]. ANT oxidative and carbonyl modifications reduce mitochondrial energy output [228,229,230,231]. The uncoupling of ATP synthesis disrupts the mitochondrial matrix disrupting Ca^2+^ stabilization [232]. In fact, failing senescent mice hearts in conditions of stress, increase by two-fold Ca^2+^ levels, increasing ischemic and reperfusion damage [233]. Progression to heart failure and heart failure itself includes various mechanisms. As seen before, oxidative stress in aging also leads to increased cardiomyocytes apoptosis, ECM remodeling, and altered response to stress all important factors for HF [85]. For all the reasons above, heart failure and aging are synonyms. The aging process and all the changes involved lead to heart failure.

### 3.3. Valvular Heart Disease

Age is the main driving factor for valvular myxomatous degeneration and valvular sclerosis. Elderly patients have a prevalence of 30–80% of aortic valve sclerosis [234,235,236]. With age echocardiographic examination in these patients shows an increase in calcification of the aortic valve leaflets and anulus [237,238]. Elderly patients also have concomitant risk factors for rapid progression of valve degeneration such as hypertension, LVH, hyperlipidemia, and kidney failure [239]. Moreover, there is a linear correlation between aortic valve degeneration and atherosclerosis in elderly patients [240]. Valve sclerosis does not have a hemodynamic impact but can evolve into stenosis. Aortic stenosis is characterized by the reduction in the opening of the valve leaflets with consequent increased in pressure gradient between the left ventricle and the aorta [239]. To guarantee the necessity of the increased pressure gradient the heart undergoes myocardial hypertrophy to maintain an adequate systolic function and cardiac output [235]. This mechanism of compensation leads to left ventricle dilatation and deterioration of systolic function on the long run. Another aortic valve degeneration consequence is aortic regurgitation, a situation where the leaflets do not close properly and a backward blood flow in generated. This second condition causes a rapid diastolic filling and eccentric hypertrophy of the heart in order to increase the blood volume output [235]. Same is true for the mitral valve, with aging there is degenerative process involving the annulus and the leaflets [241,242]. In this case, however, valve regurgitation is more common than stenosis [243]. As for the aortic valve, hypertension, kidney failure, and aortic alterations are risk factors mitral calcification and patients with this such alteration have an increased risk for heart failure, atrial fibrillation, stroke, coronary artery diseases, and overall adverse cardiovascular events and mortality [234]. In humans, mitral valve regurgitation is mainly associated with ischemic heart disease and myxomatous degeneration while mitral stenosis is associated with rheumatic disease [244]. Mitral and aortic valve vices are one of the most common cause of surgery in older population [245]. Interesting are the recent discoveries that associated reduction in telomere length with degenerative valve disease. In particular elderly patients with aortic stenosis had a reduction in leukocyte telomere length [246]. Although more research is required to fully understand ROS-induced damage to telomeric DNA, studies suggests that this may be an important factor to take into consideration [247]. Furthermore, even if oxidative stress and telomere shortening in humans still have not been directly correlated, there is a great association with frailty typical of the elderly [248]. Oxidative stress and inflammation play an important role in valvular calcification. The underlining mechanisms are not fully known but local inflammation by hyperlipidemia and diabetes has been shown to be a great promoter of valve and vascular sclerosis [249]. Moreover, human studies have assessed that oxidized lipids seem to be one of the main inflammatory driving factors [250,251,252]. Increased levels of circulating oxidized phospholipid, ox-LDL and of TNF-alpha and inflammatory cells have been highlighted in patients with higher degrease of valvular degeneration [253,254]. High levels of ox-LDL increase the expression of osteogenic factors such as BMP-2, activating TLR expression [255,256]. The vicious cycle of lipid oxidation induces chemokines release and recruitment of monocytes and enhanced expression of ICAM, and MMP, factors leading to reduction NO production [257,258,259,260,261]. In conclusion, valvular heart disease is probably associated to oxidative stress in the aging heart by ROS increase with NO synthase uncoupling mediated mainly by ox-LDL expression of NADPH [262,263,264,265,266].

## 4. Aging, Oxidative Stress, and Vascular Diseases

An increasing production of reactive oxygen species is associated with a many cardiovascular diseases [8,11]. ROS family is composed of both oxygen free radicals and non-radicals [267]. As highlighted earlier in the article mitochondria are the main productor of ROS, other sources are NADPH oxidases. Other cell organelles, including peroxisome and endoplasmic reticulum, contribute to intracellular ROS production through enzymes like xanthine oxidase, nitric oxide synthase, cyclooxygenases, cytochrome P450 enzymes, and lipoxygenase. Proteins, lipids, and DNA are the primary cellular structures affected hit by ROS and RNS. The generation of ROS is a natural part of aerobic life; indeed, basal levels of ROS are necessary to exert various cellular functions, such as signal transduction pathways, defense against invading microorganisms, gene expression, and the promotion of cell growth or death [13]. Human tissues have protective measures against ROS like superoxide dismutase (SOD), catalase, peroxiredoxin, and glutathione peroxidase, and non-enzymatic compounds tocopherol/vitamin E, beta-carotene, ascorbate, glutathione, and nicotinamide [14]. Imbalance of oxidant signaling may accelerate some pathological conditions and the rate of aging. Oxidative stress and inflammatory response are strictly related with endothelial dysfunction, the key to cardiovascular disease.

**Figure 2:**
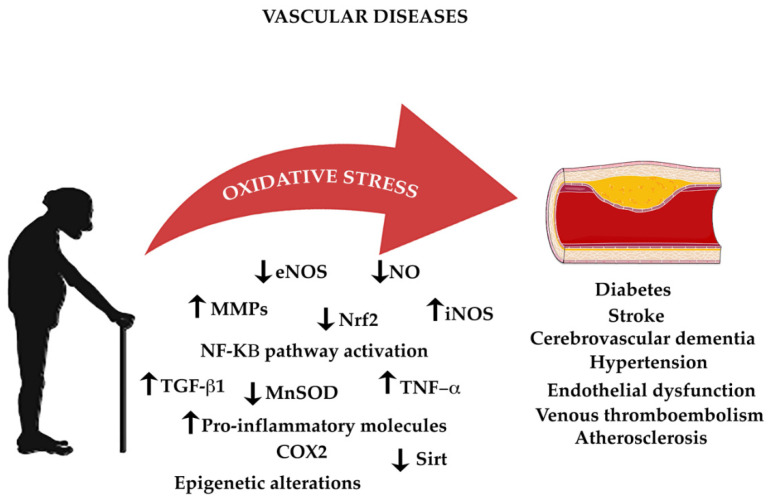
The impact of oxidative stress on age-associated vascular diseases.

The endothelium is an organ composed by a highly active monolayer of cells that modulates important functions such as vascular tone, cellular adhesion, thromboresistance, smooth muscle cell proliferation, and vessel wall inflammation. This is mediated by the release of vasodilator and contracting factors. The vasoactive molecules that relax or constrict vessels regulate tone and diameter of the vascular tree directly influencing the balance of tissue oxygen supply, long-term organ perfusion, remodeling of vascular structures, and metabolic demand [268,269]. eNOS generates the vasoprotective molecule NO, which activates soluble guanylyl cyclase and increases cyclic guanosine monophosphate (cGMP) [29]. NO can also inhibit leukocyte adhesion to the vessel wall, blocking the first step of the development of atherosclerosis [29]. Traditional cardiovascular risk factors are related with endothelial disfunction such as smoking, sedentary lifestyle, aging, hypercholesterolemia, arterial hypertension, hyperglycemia, and a family history of premature atherosclerotic disease [270]. Increased levels of superoxide anions are led by inactivation of NO. This represents a critical mechanism leading to endothelial dysfunction [271]. NADPH oxidase is a key enzyme in reducing oxidative stress and normalization of endothelial dysfunction in mice [272]. Another character related with endothelial disfunction is inflammation. In endothelial dysfunction, activation of the nuclear factor-kappa B (NF-κB) pathway has highlighted elevated levels of pro-inflammatory cytokines such as tumor necrosis factor-alpha, interleukin-1beta (IL-1 β), interleukin-6 (IL-6), and interferon gamma (IFN-γ) [273,274]. NF-κB is an important transcription factor regulating the gene expression of inflammation and redox status enzymes and playing an important role in the CVD development in humans [275]. The NF-κB pathway can be activated by a wide of stimuli including ROS, inflammatory cytokines, and mechanical forces acting on the vascular endothelial wall. The activation of a kinase (IκK) mediated phosphorylation and the degradation of the inhibitors of NF-κB (IκB) consent the translocation of the NF-κB heterodimer to the nucleus, where it binds to promoters of gene targets. Other pro-inflammatory molecules are related to endothelial dysfunction, like IL-6, TNF-α, monocyte chemoattractant protein 1 (MCP-1), and the pro-oxidant enzyme NADPH oxidase, all of which predispose the vasculature to CVD (Figure 2) [276].

### 4.1. Aging in Vascular Structural and Patho-Physiological Changes

Structural and physiological changes of the endothelium and smooth muscle cells can be detected in aging vasculature. The early manifestation of arterial aging is the impaired endothelial vasodilation that precedes in years the clinical manifestations of vascular dysfunction [277]. Another hallmark of vascular aging is an increase in arterial stiffness due to the loss of arterial elasticity, compromising vascular adaptation to blood flow and pressure changes [119]. Elastic fibers undergo fragmentation due to the enhanced MMPs activity, in fact Increased expression and activity of MMP-2 have been reported in vessels from both aged animals [278,279] and humans [280]. Alteration of morphology and composition of extracellular matrix cause an increased speed of propagation of pressure and flow waves that can be measured by different ways like the determination of the pulse wave velocity (PWV). Although MMP-2 upregulation is more commonly related to vascular aging, plasma MMP-1 and circulating markers of type 1 collagen (MMP-1 substrate) degradation positively correlate with PWV and augmentation index as indicators of arterial stiffness in human subjects [281]. The increased severity of vascular diseases observed in aging vasculature is in part due to functional decline of endothelium repair capacity observed in elderly [116]. This could be related to the decreased number of endothelial progenitor cells (EPC) and to the impairment of their functions with consequent the loss of function in mechanisms for endothelial regeneration and maintenance [117]. Several studies have demonstrated the impaired reendothelization ability of EPCs [282] and a reduced number or circulating EPCs in elderly subjects [115,283]. The first and most important alteration of aging vasculature is endothelial dysfunction, a determinant factor for macro and microvascular dysfunction, related with an elevated morbidity and mortality [101,102]. Arterial stiffness is always preceded by an impaired endothelial vasodilation, suggesting that this arterial alteration is also linked to endothelial dysfunction [113]. In particular, aging is an independent factor associated with the reduction in the endothelium-dependent vasodilatation even in the absence of other cardiovascular risk factors [28,284]. In fact, it is described both in vitro and in vivo in different on old animals [31,103] and humans [102]. NO is a molecule key to cardiovascular health produced by NO synthase isoforms, most importantly eNOS [108]. NO production seems to have an important role in longevity [285]. The mechanisms of endothelial dysfunction involve a diminished availability of NO, and an imbalance between the endothelium-derived vasodilators and vasoconstrictors. Aging is associated with deficit in NO bioavailability; however, the impact of aging on eNOS expression is controversial. eNOS expression in aged vessels could be unchanged [286] decreased [287], or increased [3,288]. In contrast, it is well accepted that eNOS activity is reduced with aging [109]. Although reduced eNOS activity possibly accounts for the decline in endothelial vasodilation with aging; other two mechanisms are responsible for endothelial dysfunction in aging: an excessive chronic oxidative stress and an increased proinflammatory activity both compromising the bioavailability of nitric oxide as discussed previously [137,289].

### 4.2. Hypertension

Hypertension is a clinical condition in which the blood pressure in the arteries of the systemic circulation is elevated. Blood pressure is summarized by two measures, systolic and diastolic, which depend on the fact that the heart muscle contracts (systole) and relaxes (diastole) between one beat and another. Hypertension is considered if there is a pressure frequently equal to or greater than 140/90 mmHg [290]. Hypertension is classified as primary (essential) or secondary. About 90–95% of cases are classified as “primary hypertension,” which means there is high blood pressure with no obvious underlying medical cause. The remaining 5–10% of cases classified as “secondary hypertension” are caused by other diseases affecting the kidneys, arteries, heart, or endocrine system [291]. Hypertension is a risk factor for stroke, myocardial infarction, heart failure, arterial aneurysms, peripheral arterial disease and is a cause of chronic kidney disease [292]. Even moderate elevations in arterial blood pressure are associated with a reduction in life expectancy [293]. Hypertension is the largest contributor to the global burden of CVD and is for many states a heavy burden on the socio-economic expenses [294]. Both aging and hypertension are associated with morphological and functional changes in the vascular tree characterized by increased arterial stiffness, reduced elasticity, impaired distensibility, endothelial dysfunction, and increased vascular tone. The relationship between aging and vascular stiffening is evident in patients with progeria (premature aging), who exhibit accelerated vascular aging and often die of cardiovascular disease [295]. Findings from noninvasive vascular phenotyping studies in healthy individuals have demonstrated that intima-media thickness increases 2- to 3-fold between 20 and 90 years of age [118] The precise factors causing progressive intimal thickening with aging is still not clear, but a number of distinctive changes at cellular and morphologic levels have been identified: increased collagen deposition, cellular senescence, unfit elastin fibers turnover, and dysregulated cell proliferation. The remodeling of the ECM, which is an essential component of the connective tissue surrounding the vascular wall, has a key role in vascular aging. The ECM is composed of structural elements like collagen and elastin and more specialized proteins including fibronectin and proteoglycans. The ECM is a dynamic structure regulated by MMPs and TIMPs. Dysregulation of these processes, together with alterations in profibrotic and proinflammatory pathways, contributes to structural modification occurring with aging. Both large and small arteries are interested by fibrosis. In large vessels, vascular stiffening leads to hemodynamic damage to peripheral tissues [296]. Aortic wall stiffening causes increased pulse wave velocity (PWV) and premature reflected waves with elevated central hemodynamic load leading to damage of peripheral small arteries [297]. Arterial stiffness can be evaluated by measuring PWV, measuring the speed of the pressure pulse from the heart propagated to the arteries. This can be calculated by relating the distance travelled and the time taken to travel a defined distance. Stiffer arteries increase time travel resulting in higher PWV. Carotid-femoral PWV is the gold standard for measuring aortic stiffness [298]. Arterial stiffness has a bidirectional causal relationship with blood pressure because high blood pressure causes arterial wall injury, which in turn promotes stiffening. This is the main mechanism of increasing systolic blood pressure in aging vasculature [299,300]. A lot of evidence has shown an important relationship between oxidative stress, inflammation, and hypertension [301,302]. Oxidative stress is the main character of vascular inflammation and the consequent inflammatory responses [303,304]. Immunosenescent T cells have an important role in hypertension and the susceptibility of T cells to changes associated with aging like by loss of CD28 marker and appearance of CD57 [305]. They produce tumor necrosis factor-α and interferon-γ which affect vascular changes [306]. T cells are key in vascular disease [307,308] and several clinical studies indicate that their role is due to a close interaction with other elements of innate, cellular, and humoral immunity [307]. B cells also seem to be essential in hypertension, as their lack is related with a reduced vascular oxidative stress [309]. In fact, B cells may explicate their effects with antibodies and with cytokine production like TNFα, which has been shown to be prohypertensive and pro-atherosclerotic [310]. By the same rational, macrophages also have an evident role in hypertension and atherosclerosis [311,312] closely linked to NADPH oxidases abundance in their cytosol [313]. NADPH oxidase also participates in vascular damage, inflammation, and fibrosis [314,315]. TNFα acts as a pro apoptotic factor stimulating ROS production. This mechanism promotes accelerated vascular aging [316] as well as cognitive impairment [317]. Inflammation and oxidative stress are important for microvascular dysfunction. This has particularly relevance in conditions associated with small vessel disease and cognitive impairment. In fact, hypertension has been shown to exacerbate Alzheimer disease through reduced NO bioavailability trough eNOS and nNOS activity [318]. Important microvascular dysfunction leading to cognitive impairment is dependent on NADPH oxidase activation in macrophages infiltrating perivascular space in the brain [317]. This seems to be a mechanism for NO decrease and neurovascular unit impairment [317] demonstrating the role of inflammation in cognitive dysfunction. The interaction between inflammatory response and oxidative stress influence vascular remodeling through mediators like MMPs, which degrade collagen and elastin and promote fibrosis mechanisms. Their inhibition retards arterial remodeling decreases stiffness and improves endothelial function in animal models [319]. This is because MMPs create an inflammatory condition that contributes to shift the phenotypes of endothelial cells and smooth muscle media cells toward secretory and senescent phenotype which in turn increase MMP levels in vasculature. The effects of MMPs are mediated by ROS [320]. In fact, MMP genes are redox sensitive and are regulated by increased NADPH oxidase activity [321]. In summary MMPs promote arterial remodeling in aging, hypertension, and atherosclerosis [322]. Therefore, tissue inhibitors of metalloproteinases, such as TIMP-2, play protective role like suppressor of vascular remodeling of the ECM [323]. In summary, the key to vascular aging is the senescence of the vascular wall cells which promotes chronic ECM degradation and perivascular inflammation. Multiple factors contribute to vascular aging and hypertension. In aging vasculature, increased levels of Ang II [324], angiotensin-converting enzyme [325,326,327], mineralocorticoid receptors [328], and endothelin-converting enzyme-1 have been highlighted [329,330], contributing to structural and mechanical changes in the vascular tree. In particular by activation of AT-1 receptors Ang II AT1 plays a major role in the production of ECM proteins [331,332]. This was demonstrated by studies where the antagonism of Ang II receptors resulted in decreased fibrosis [333,334]. The precise signaling events involved in Ang II-induced vascular fibrosis is still not clear but is known that activity is increased by Ang II [331]. TGF-β1 is expressed in endothelial cells, vascular smooth muscle cells, myofibroblasts, and adventitial macrophages. Activation of vascular TGF-β1, and its downstream signaling effector SMAD results in excessive matrix accumulation, in part due to the inhibition of ECM degradation [335]. Several studies highlighted aldosterone as an important pathophysiological mediator in cardiovascular remodeling promoting vascular hypertrophy, fibrosis, inflammation, and oxidative stress [336,337]. In fact, chronic blockade of mineralocorticoid receptors, through which aldosterone signaling works, showed reduction of cardiovascular fibrosis. This effect may be due to the increasing collagen I synthesis [338,339]. Another vascular factor that influences the hypertension phenotype is endothelin (ET). The vascular actions of ET are mediated by 2 distinct endothelin receptor subtypes: ETA and ETB receptors located on vascular smooth muscle and on endothelial cells. ET modulates ECM stimulating fibroblast-induced collagen synthesis and acts as a hypertrophic and mitogenic factor. ET-1 stimulates synthesis of collagen through both ETA and ETB receptor subtypes [340,341]. Moreover, arterial stiffening derives from imbalance between excessive fibrosis due to excessive accumulation of vascular collagen and degradation of elastin. It is a dynamic phenomenon and one of the main markers of vascular aging in hypertension and other cardiovascular diseases.

### 4.3. Atherosclerosis

Atherosclerosis (AS) is a chronic disease of the arterial wall [342] and one of the most common cause of CVDs [343], which widely affects public health in our society. Specifically, the accumulation and rupture of atheromatous plaques in arteries can lead to ischemic heart disease, ischemic stroke, and peripheral arterial disease [342]. The disease has a latency of many years and frequently coexists in more than one vascular bed [344]. In particular, the central arteries undergo substantial structural and functional alterations with aging: they dilate, leading to an increase in lumen size, while intima becomes thickened, and the media exhibits an increased collagen content and frayed elastin [345,346]. The thickness of the arterial wall, which arises mostly in the intimal layer, increases two-to three-fold between 20 and 90 years, consequently increasing the risk of AS [118,345]. Furthermore, the intimal medial thickness is a marker of subclinical vascular disease: a 0.1 mm increase in carotid artery intimal medial thickens is associated with an 18% increase for stroke and 15% for myocardial infarction [347]. In particular, AS is a peculiar form of inflammation triggered by cholesterol-rich lipoproteins and other noxious factors such as blood pressure, cigarette smoking, diabetes mellitus, and chronic kidney disease [348]. However, genetics also play an important role in the disease, accounting for about 40% of the risk [348]. Briefly, AS is a multifactorial and progressive disease. In fact, there is an emerging indication that inflammatory pathways play a crucial role in atheroma construction. Stimulation of pro-inflammatory signaling pathways, expression of cytokine/chemokine, and increased oxidative stress are some of the pathways leading to AS [11]. Noxious factors lead to qualitative changes in endothelial cells, encouraging the expression of adhesion and chemotactic molecules and an amplified permeability to macromolecules [6]. This enables the infiltration of subendothelial spaces of arteries by oxidized lipoprotein. This leads to the formation of plaques, cholesterol deposits (atheroma) with a fibrous cap (sclerosis), characterize the inflammatory process of AS [79]. The aging process could accelerate structural and compositional changes observed in AS [79]. As known, endothelial cell injury and AS suggest the susceptibility of aged vessels to the lesion [349]. Previous studies compared old and young rabbits subjected to a long period of hyperlipidemic diet: old rabbit arteries constantly develop fibroatheromatous plaques [350]. Some studies have addressed the implications of cellular senescence in the AS pathway [351]; specifically, the cellular senescence could occur in two forms: replicative and stress-induced premature senescence [352]. The replicative pathway is due to DNA damage-induced telomere shortening, which could result from the high content of reactive oxygen species (ROS) and oncogenes [79]. Furthermore, biomarker such as senescence-associated β galactosidase (SAβG) was found in senescent human cells [353]. A high amount of SAβG-positive in atherosclerotic lesions and old vessels reinforced the association between atherosclerosis and senescence [354]. ROS are the major candidates responsible for senescence and age-related diseases in which the redox balance is invalidated and generates oxidative stress [355]. Age-related oxidative stress is essential to the pathologic changes of AS, because the generation and clearance of oxidative stress in the vasculature is a significant regulator. On one hand, endothelial cells and vascular smooth muscle cells produce reactive oxygen and nitrogen species, which oxidize low-density lipoproteins (LDL); moreover, the oxidation process increases mitochondrial rupture and the release of proapoptotic molecules to the cytosol, increasing plaque cell apoptosis [356]. On the other hand, the scavenging process for reactive nitrogen species increases endothelial dysfunction, smooth muscle cell proliferation, leukocyte adhesion, and inflammatory responses [357]. Some research on AS have linked nutrition to age-induced vascular changes [358]; specifically, caloric restriction (CR) could be a dietary intervention for promoting longevity and delaying age-related diseases, such as AS [359]. Kitada and colleagues [359] demonstrated that CR-induced Sirt1 has an antiaging property, with many physiological benefits: reduced apoptosis, enhanced mitochondrial biogenesis, inhibition of inflammation, regulation of glucose and lipid metabolism, and adaptations to cellular stresses. Further, observational data collected in humans show that the ingestion of antioxidants is associated with prevention of cardiovascular disease [360] and intrinsic antioxidant systems in the mitochondria could be therapeutic targets [355]. On the other hand, exercise is known to reduce the risk of cardiovascular disease. In particular, the beneficial effect of exercise on improvement of serum atherogenic lipoprotein profiles include reduction of serum triglycerides, increase in HDL cholesterol, and increase in average size of low-density lipoprotein (LDL) particles [361]. Furthermore, higher physical activity has also been shown to reduce carotid artery intima-media thickness, a clear marker of atherosclerotic burden [85].

### 4.4. Deep Vein Thrombosis

Venous thromboembolism (VTE) refers to the interconnected diagnoses of deep vein thrombosis (DVT) and pulmonary embolism (PE). Specifically, DVT is the formation or presence of a thrombus in the deep veins [362]. VTE is globally the third most frequent acute cardiovascular syndrome behind myocardial infarction and stroke [363], and thromboembolic events count among the most common complication of hospitalization [364]. VTE is a significant cause of mortality and disability, affecting 1–2 per 1000 people annually, presenting with a rather wide range of symptoms, which can pose a diagnostic challenge [365]. Particularly, DVT and PE account for 60,000 to 100,000 deaths annually in the United States [366], while in Europe, this number rises to greater than 500,000 deaths annually [364]. Considerable research has been done to identify risk factors and to provide appropriate prevention where indicated. However, VTE remains a growing public health concern [364].

Venous thrombosis is the development of a platelet and fibrin clot in the vascular lumen. Thrombosis formation is a dynamic, multicausal process that hinges on a fine balance of physical and biochemical features [366]. In particular, DVT is considered to be the result of the interaction between patient-related (usually permanent) risk factors and setting-related (usually temporary) risk factors [367]. According to Virchow’s triad, the pathophysiological mechanisms involved in DVT are three: damage to the vessel wall, blood flow turbulence, and hypercoagulability [368]. Clinically significant thrombi are shaped in vessels with large lumens such as the deep veins of the legs, pelvis, and arms; however, the clot can then propagate with proximal extension [364]. Moreover, if the clot dislodges, it can embolize to a distant site, and the most common site of embolization is in the pulmonary vasculature [364].

Aging is associated with an increased risk for thrombosis. It has been documented that the incidence of VTE is two to seven times higher in patients above the age of 55 as compared to a younger cohort [369]. Specifically, several factors related to aging have been observed: increased frequency of illness and periods of prolonged immobility, greater prevalence of obesity, comorbidity, and an increase in the level of procoagulants without a commensurate increase in anticoagulants [370]. Rumley and colleagues [371] demonstrated that the increase in risk of VTE in men between age 60 and 79 years may be related in part to increased activation of blood coagulation, fibrinolysis, and inflammation. They highlighted a marked increase in fibrin D-dimer and CRP in this population as relevant to the pathogenesis of VTE with increasing age. On the other hand, aging is associated with increased production of ROS, which indirectly creates a discrepancy between thrombosis and hemorrhage [372,373].

Up-regulation of ROS-producing enzymes (e.g., NADPH oxidase and myeloperoxidase), along with down-regulation of antioxidant enzymes (e.g., SOD and glutathione peroxidase GPx), occur in the elderly. This imbalance may lead to thrombosis by damaging red blood cell (RBC) shape and function, causing endothelial dysfunction, and triggering platelets and leukocytes, therefore affecting the clotting system [374]. RBCs can be a major source of oxidative stress in elderly, since RBC redox homeostasis is usually compromised. In fact, ROS con easily accumulate within the RBC due to constant autoxidation of endogenous hemoglobin (Hb) and to NADPH oxidase activation. Moreover, RBC in their path uptake extracellular ROS released by other cells in the circulation [374]. Specifically, ROS affect the RBC membrane structure and function, causing loss of membrane integrity, and decreased deformability [374]. This process impairs RBC function in hemostasis and thrombosis, supporting a hypercoagulable state. Overall, these changes lead to enhanced RBC aggregation by increased binding to endothelial cells which also affects nitric oxide availability [375]. Accelerated aging and hemolysis is also a key factor as shown by increased RBC-induced platelet activation, RBC interaction with coagulation factors, increased RBC phosphatidylserine exposure and release of microvesicles [374].

In summary, aging and oxidative stress influences greatly RBC deformability, blood rheology, altering blood flow in the circulation [376]. All the above combined with genetic polymorphisms and mutations can trigger venous prothrombotic events [377], thus predisposing the elderly to age-related VTE [374]. In conclusion, a further better understanding of the detailed mechanisms by which aging, and the associated oxidative stress disrupt the coagulation cascade to initiate venous thrombosis would provide new targets for tailored therapies to prevent VTE in the elderly.

### 4.5. Stroke, TIA, and Cerebrovascular Dementia

A stroke or cerebrovascular accident is an acute compromise of the cerebral perfusion or vasculature [378]. Almost 85% of strokes are ischemic and the rest are hemorrhagic [379]. On the other hand, a transient ischemic attack (TIA) is defined as a transient episode of neurologic dysfunction due to the focal brain, spinal cord, or retinal ischemia, without acute infarction or tissue injury [380]. Over the past several decades, the incidence of stroke and mortality is decreasing; however, the burden of stroke in the US remains enormous and stroke is the second most frequent cause of death after ischemic heart in developed countries [381]. Furthermore, this disease commonly occurs in individuals over 65 years [382] and is the leading cause of adult disability worldwide [378]. Moreover, the estimated overall prevalence of TIA among adults in the US is about 2% [383]. Ischemic stroke can result from embolic, thrombotic, and lacunar events. Generally, the common risk factors for stroke include hypertension, diabetes, smoking, obesity, atrial fibrillation, and drug use. Hemorrhagic etiologies can result from hypertension, arteriovenous malformations, aneurysm rupture, venous angiomas, amyloid angiopathy, and other obscure etiologies. Of all ischemic strokes, lacunar strokes represent 20% and results from occlusion of the small penetrating branches of the middle cerebral artery, vertebral or basilar artery, or the lenticulostriate vessels [378].

Atherosclerosis is the most significant underlying pathology in stroke and leads to the formation of atherothrombotic plaques in the arteries supplying the brain [384]. Plaque rupture causes exposure of the underlying cholesterol crystals which attract platelets and fibrin, and formation of fibrin-platelet-rich emboli leads to stroke in the distal territories [384].

In summary, stroke is the result of ischemia in an area of the brain. When there is a break of blood supply by a thrombus, the immediately adjacent neurons lose their supply of oxygen and nutrients. The inability to use aerobic metabolism and produce ATP causes the Na+/K+ ATPase pumps to fail, causing an accumulation of Na+ inside the cells and K+ outside the cells [384].

Consequently, ischemia leads to depolarization of cells which results in calcium influx into cells, elevated lactic acid, acidosis, and free radicals’ formation [385]. Cell death increases glutamate and leads to a cascade of chemicals (excitotoxicity) [378,385].

It has been reported that the incidence of stroke increases with age [386] and numerous researches have proved that aging or senescence is a risk factor that exacerbates stroke. Moreover, oxidative stress is certainly connected to age-associated cognitive decline [387].

Specifically, a recent study revealed that both amplified oxidative stress biomarkers connected with raised levels of inflammatory cytokines were related to poor cognitive performance in elderly people [41,388]. At midlife, neuronal atrophic and glial cell changes begin at the same time with degeneration of white matter. Consequently, aging-induced alterations of white matter can influence the susceptibility of axons to ischemia. The aging process influences the brain microvasculature by a great extent. The structural and functional degeneration of the blood-brain barrier (BBB) during aging could disrupt local blood perfusion and nutrient supply [79].

Cerebral vessel modifications in the elderly may decrease cerebrovascular reservoirs and make the brain more prone to ischemic damage and vascular insufficiency [389]. All these alterations could lead to an ischemic stroke and vascular cognitive deficiency in aging.

In vascular inefficiency, ROS and reactive nitrogen species (RNS) cause an increased concentration of H+ and H_2_O_2_, leading to DNA injury, endothelial alteration, and mitochondrial dysfunction [390]. Moreover, this is worsened by damage through ROS-mediated inflammation, apoptosis, autophagy, and the microbial-gut-brain axis [390]. The depletion of energy in ischemic stroke can cause a series of injuries to promote the progress or recurrence of stroke, and OS is involved in all phases of ischemic stroke evolution. As reported, energy expenditure leads to the accumulation of H+ concentration and H_2_O_2_. Then, ROS increases vasoconstriction and triggers platelet aggregation and endothelial cell permeability, affecting blood circulation [391]. Additionally, RNS plays a role in mitochondrial functions, for instance, by reducing DNA and suppressing enzymes of the mitochondria [390]. Finally, ROS and RNS bring about DNA damage, protein destruction, lipid peroxidation, and cell death, leading to poor outcomes [390,392]. As expected, the results of clinical studies have confirmed that higher plasma levels of oxidized low-density lipoprotein led to a worse prognosis [393], an increased risk of death [394] and a higher prevalence of cognitive impairment [395].

Finally, there are important molecular and signaling pathways modifications with aging [390]. Under ischemia/hypoxia conditions in the elderly, Sirt1 expression and the mitochondrial unfolded protein response decrease in aging, leading to poor mitochondrial function and fitness [396]. This condition leads to an inadequate post-translational regulation of molecular mediators, such as hypoxia-inducible factor 1α and Sirt1, and of the glycolytic-mitochondrial energy axis, all fundamental in response to hypoxic-ischemic injury [397]. In summary, OS has a central role in the “aging-stroke” system. First, when a stroke occurs in animals or patients, there is an excessive generation of ROS, causing cellular damage and brain injury [390]. Second, OS mediates inflammation, apoptosis, and the microbiota-gut-brain axis to increase the accumulation of ROS, followed by brain alteration [390]. Third, aging is a risk factor and exacerbates the progress of stroke via OS and OS-induced pathways [390]. Consequently, the administration of antioxidants could provide therapeutic ways for stroke. Indeed, the use of antioxidants in animals has been successful in treating stroke [398]; furthermore, several clinical trials show that co-antioxidants exert protections in stroke, including flavonoid and melatonin [399,400]. Moreover, clinical trials show that also acupuncture stimulates the inherent antioxidant enzyme system and inhibits the disproportionate generation of ROS, by regulating a series of molecular signaling pathways in redox modulation [401]. Acupuncture therapy has the potential in alleviating oxidative stress caused by cerebral ischemia, which may be associated with the neuroprotective effect of acupuncture [401]. In conclusion, reducing stroke frequency by preventive measures, as well as cerebrovascular dementia, is essential to avoid the natural trend of increase in the human, economic and social burden of these diseases.

## 5. Possible Antiaging and Antioxidant Interventions

Thanks to the technological advancements in medicine many research groups have tried to find therapeutical options for aging and its correlated effects like oxidative stress.

A well-established strategy against aging is dietary restriction (DR). This intervention has been shown to increase life span and decrease the onset and progression of age-relates pathologies, like the ones discussed in our review. Although the true mechanisms behind these beneficial effects are still to be fully grasped, many model organisms agree [402,403,404,405,406]. In particular, dietary restriction in rodents and non-human primates has shown to have cardiac preventing properties [403,407,408]. This beneficial pattern has been also confirmed in humans by trials using alternated day fasting [409]. The activation of the pathways associated with dietary restriction overlap those associated with exercise training including mTOR inhibition and Sirt activation [410,411,412]. These two pathways are strongly NO signaling associated [413,414]. These findings are backed by an increasing number of studies on cardiac aging. In fact, nutrient mediated signaling by DR influences the heart by affecting cardiac stem cells, mitochondrial efficiency, and ROS production [407,415,416]. The use of DR in clinical settings are still to be sufficiently schematized. Since the beneficial effects of DR on preventing CVD and on cardiac aging are undoubted, future studies must investigate on the application of this therapeutic strategy.

As seen throughout our review oxidative stress plays a crucial role in CVDs. For this reason, many studies have suggested the possible therapeutic application of antioxidants. Ten of thousands clinicals trials have evaluated antioxidants effects on CV events and mortality but results so far have been controversial [417,418]. Among antioxidants, vitamins have shown some effect in short term secondary CV prevention as in the case of vitamin E and C, although long term follow-up was non encouraging in humans [417]. Even more controversial is the supplementation with Vitamin A, which even worsened outcome in all-cause mortality [419,420]. Taken this disappointing outcome in vitamins use, there are still many other types of antioxidant agents and there are many potential reasons for the failure of these clinical trials [421]. Unfortunately, it is as of now impossible to use endogenous antioxidant enzymes, SOD, catalase or GPx, or any synthetic deriving molecule. Several attempts have been made to create such molecules, mimetics and or scavenging enzymes, but so far, no elements have been successful, and other molecules are still in trial [422,423,424,425,426].

Antioxidant elements of great interest in the last year for their many properties are nutraceuticals [427,428]. Nutraceuticals are nutrients contained in foods that have beneficial effects on health. They are found in nature and can be extracted, used for food supplements, or added to food as it is rare to find them in sufficient quantities to obtain benefits in natural foods [429]. Nutraceutical foods are also commonly referred to as functional foods. These are such if the food naturally contains the nutrients in the minimum quantities to obtain beneficial effects. Often the latter are obtained through chemical syntheses. Nutraceuticals are normally derived from plants, foods, and microbial sources [430]. Examples of nutraceuticals are probiotics, polyunsaturated fatty acids (omega-3, and omega-6), vitamins, and enzyme complexes. They are typically used to prevent chronic disease, improve health, delay the aging process, and increase life expectancy [431]. Nutraceuticals can be taken either in the form of a “naturally nutraceutical food” or as a “food enriched” with a specific active ingredient (for example, milk with added vitamin D or omega-3 acids). They can also be taken in the form of food supplements in liquid formulations, in tablets, capsules, and in isolated or combination [428,432]. The wide range of these molecules and the lack of knowledge in the single and combined effects make it difficult to have a precise consideration of the therapeutic strategy to implement in clinical settings [433,434]. Among these, resveratrol and Sirts activation as a DR-mimetic have elicited great research interest. Resveratrol is a compound found in grapes and wine and has been associated with many cardioprotective effects including antioxidant, cardiac anti-aging, antiaggregating, enhance eNOS signaling effects, and LDL-ox reduction effects [433,435,436]. Worth noting is resveratrol’s effect on cardiac diastolic dysfunction, in insulin resistance, and on transcriptional profile [437].

As seen before, the renin-angiotensin system (RAS) and the autonomic nervous system (ANS) play an important role in development of CVD. In particular, hypertension is the main disease deriving from these systems alteration. Angiotensin-converting enzyme inhibitors (ACE-i), which inhibits Ang-II formation, and AT-1 receptor blockers (ARB), that blocks directly Ang-II receptor activation, have been extensively used and have shown not only anti-hypertensive properties but also antioxidant ones [438]. Ang-II is a potent vasoconstrictor and pro-inflammatory factor [439]. It has been shown to activate and recruit monocytes and macrophages into the vascular wall acting as pro-fibrotic agent [440]. Therefore, basic clinical evidence has shown that attenuation of Ang-II effects leads to immediate reduction of arterial blood pressure, but some beneficial effects of ARB and ACE-I are independent from this aspect [441,442]. Inhibition of RAS is beneficial even when it is not at the stem of hypertension [443]. Thus, independently from hemodynamic effects ACE-I and ARB can exert beneficial tissue effect. In particular, ACE-I is also involved in accumulation of bradykinin which induces release of nitric oxide (NO), prostacyclin and endothelium hyperpolarizing factor [444]. By the same logic, ARB can also stimulate bradykinin, NO, and prostaglandin release [445]. Thanks to their activity ACE-I and ARB can reduce superoxide generation, increasing NO bioavailability [446,447]. Mainly, in vitro studies on captopril, enalapril and lisinopril have highlighted ACE-I scavenger properties to contrast free radicals [448]. However, not all reports agree and some highlight that only sulfhydryl ACE-I have this property and that even these may not have in vivo effect due to low tissue concentration in normal therapeutic use [449,450]. Nevertheless, studies on mice with non-hypotensive doses of ACE-I for 11-weeks increased antioxidant enzymes and glutathione content in tissues [451,452]. Interestingly, the erythrocytes of these mice were more resistant to oxidative stress [453]. Lastly, these drugs also elicited positive effects against oxidative stress in streptozotocin-induced diabetic rats [451].

Worth mentioning are mTOR inhibitors agents, like rapamycin (Sirolimus) and Everolimus [454]. These gerosuppressant drugs have been shown to slow down the aging process and extend the life span of different species [455]. Inhibitors of mTOR from worms to mammals can prevent age-related diseases like the ones we have discussed in this review [456]. Before diving in, we must remember that mTOR is a signaling pathway integrated by two protein complexes, mTORC1 and mTORC2 [457]. These two complexes differ in regulatory proteins as the first is regulated by Raptor while the second by Rictor [458]. mTOR and WNT pathways work in concert to guarantee cardiac and endothelial cells life cycle [459,460,461,462]. Overall, mTOR function has been seen to regulate proliferation/differentiation of embryonic stem cells, maintaining pluripotency of embryonic stem cells when needed [463,464]. It regulates apoptosis and autophagic cell death modulating programmed cell death [465,466]. Mice models have highlighted how mTOR can protect cardiomyocytes against ischemia/reperfusion oxidative damage and can prevent cardiac atrophy inhibiting cardiomyocytes autophagy [456,467,468,469,470,471]. Similar models have also shown how mTOR can promote proliferation of endothelial progenitor cells and endothelial cells promoting angiogenesis as well as preventing induction of matrix metalloproteinases [457,460,472,473]. Rapamycin has been used in healthy volunteers with minimal side effects to investigate its anti-aging properties [474]. Its main application so far has been in patients with transplants. Fortunately, this application has made and will allow extensive research on human subjects [475,476]. The vital regulatory pathways of mTOR as still to be completely discovered but its new therapeutic beneficial effects are so far quite fascinating [468].

The latest discoveries in the frontier of senolytic agents involve chimeric antigen receptor (CAR) T cells [477]. As extensively described before chronic accumulation of senescent cells, oxidative stress, fibrosis, and many other factors contribute to the onset of age-correlated diseases [478]. Elimination of any of these factors could be the next step to finding therapeutic strategies against aging and aging diseases [479]. To this regard, CAR-T cells have been engineered to target senescent cells in disease models. To achieve this, Amor et al. engineered CAR-T cells that could target urokinase-type plasminogen activator receptor (uPAR). This senescent-specific cell-surface protein is upregulated in senescent cells both in vivo and in vitro [480]. Amor et al. [481] have used these cells in an array of senescence-induced disease models such as lung adenocarcinoma and carbon tetrachloride and non-alcoholic steatohepatitis (NASH)-induced liver fibrosis [481,482]. The yielded results are truly promising both for efficacy and safety profile [481]. The range of therapeutic application of this new technique is broad and as of now engineered T cells are at test against cardiac fibrosis and other aging correlated diseases [483,484]. A table summarizing the trials aimed at reducing cardiovascular aging is provided (Table 1).

## 6. Conclusions and Future Perspectives

CVDs are the leading causes of death in industrialized countries of the western world and aging is one of the most significant risk factors. These countries are characterized by a constant increase in the elderly population which by 2030 is predicted to become more than 20% of the population [485]. The rapid increase of the aging population highlights the necessity to develop new and better therapeutic molecules and strategies in order to prevent, treat and better overall outcome of CVDs [3]. In order to face up to this objective it is mandatory to prevent in elderly myocardial dysfunction, left ventricle hypertrophy, diastolic dysfunction, hypertension, and all those factors that we have previously described. Addressing these aging and oxidative stress induced pathologies is fundamental in order to better cardiovascular and overall outcome [486]. Many steps are still required to fully understand all the processes linked to aging and to oxidative stress related modifications in CVD in order to prevent, better, or delay cardiovascular decay. Studies are required at all levels to reach such an ambitious goal. In particular, clinical trials are still at the beginning and although some anti-aging and antioxidant molecules have had a disappointing start, it is important to keep clinical trials going. Finding new molecules and new ways to address aging and oxidative stress is fundamental to find new potential application for cardiovascular prevention and treatment.

## Figures and Tables

**Table 1:** Summary results of intervention studies exploring the effect of antiaging and antioxidant strategies.

Species	Intervention	Results	Reference
Mice	Caloric restriction	Protection of mitochondria in aging heart	Shinmura et al. [414]; Hepple et al. [415]
Mice	Resveratrol or caloric restriction	Prevention of age-related cardiac dysfunction	Barger et al. [437]
Mice	Nutraceutical combination	Reduction of blood pressure and oxidative stress, improvement of endothelial function and increase of nitric oxide release	Carrizzo et al. [428]
Mice	Enalapril and captopril	Enhancement of endogenous antioxidant defenses in mouse tissues	de Cavanagh et al. [452]
Mice	CAR-T cell	Elimination of senescent cells and extended survival in lung adenocarcinoma	Amor et al. [481]; [482]
Rats	Enalapril	Reduction of oxidative stress in diabetic rats	de Cavanagh et al. [451]
Rats	Caloric restriction	Improvement of cardiac aging and diastolic dysfunction in the senescent myocardium	Shinmura et al. [408]; Niemann et al. [407]
Rhesus monkeys	Caloric restriction	Delay in age-associated pathologies incidence (diabetes, brain atrophy, cancer, cardiovascular disease) and mortality	Colman et al. [402]; Fowler et al. [404]; McKiernan et al. [406]
Humans	Nutraceutical combination	Reduction of blood pressure, increase of nitric oxide release, and improvement of exercise capacity in hypertensive patients	Carrizzo et al. [428]
Humans	Caloric restriction or alternate-day fasting	Cardiovascular aging and chronic disease protection	Cruzen et al. [403]; Varady et al. [409]
Humans	Polyphenols	Reduction of systolic and diastolic blood pressure in high cardiovascular risk population	Medina-Remón et al. [425]
Humans	Dietary supplements, fish oil, green tea extract, resveratrol, vitamin E, vitamin C, and tomato extract	Improvement of endothelial function, reduction of inflammation and oxidative stress	Bakker et al. [426]; Schwingshackl et al. [434]
Humans	Resveratrol	Reduction of oxidative stress and endothelial dysfunction in hypertensive patients	Carrizzo et al. [436];
Humans	Rapamycin	Reduction of senescent markers in patients with coronary artery disease	Singh et al. [475]; Krebs et al. [476]
